# The feedback between selection and demography shapes genomic diversity during coevolution

**DOI:** 10.1126/sciadv.aax0530

**Published:** 2019-10-02

**Authors:** Cas Retel, Vienna Kowallik, Weini Huang, Benjamin Werner, Sven Künzel, Lutz Becks, Philine G. D. Feulner

**Affiliations:** 1 Department of Fish Ecology and Evolution, Center for Ecology, Evolution and Biogeochemistry, EAWAG, Swiss Federal Institute of Aquatic Science and Technology, Kastanienbaum, Switzerland.; 2 Division of Aquatic Ecology, Institute of Ecology and Evolution, University of Bern, Bern, Switzerland.; 3 Community Dynamics Group, Department of Evolutionary Ecology, Max Planck Institute for Evolutionary Biology, Plön, Germany.; 4 Group of Theoretical Biology, The State Key Laboratory of Biocontrol, School of Life Science, Sun Yat-sen University, Guangzhou, China.; 5 Complex Systems and Networks Research Group, School of Mathematical Sciences, Queen Mary University of London, London, UK.; 6 Evolutionary Genomics and Modelling Lab, Centre for Evolution and Cancer, The Institute of Cancer Research, London, UK.; 7 Department Evolutionary Genetics, Max Planck Institute for Evolutionary Biology, Plön, Germany.; 8 Kiel Evolution Center, Biologiezentrum, Kiel, Germany.

## Abstract

Species interactions and coevolution are integral to ecological communities, but we lack empirical information on when and how these interactions generate and purge genetic diversity. Using genomic time series data from host-virus experiments, we found that coevolution occurs through consecutive selective sweeps in both species, with temporal consistency across replicates. Sweeps were accompanied by phenotypic change (resistance or infectivity increases) and expansions in population size. In the host, population expansion enabled rapid generation of genetic diversity in accordance with neutral processes. Viral molecular evolution was, in contrast, confined to few genes, all putative targets of selection. This study demonstrates that molecular evolution during species interactions is shaped by both eco-evolutionary feedback dynamics and interspecific differences in how genetic diversity is generated and maintained.

## Teaser

Rapid genomic changes during a coevolutionary arms race highlight the reciprocal effects of ecology and evolution.

## INTRODUCTION

Host-pathogen interactions are commonplace and play a major role in many ecological and evolutionary processes (*1*, *2*), affecting the pace of molecular evolution (*3*) and rates of diversification and speciation (*4*). When species coevolve, the selection they impose on each other is temporally variable (*5*). Coevolutionary dynamics of hosts and parasites are traditionally placed on a spectrum between two extremes, where evolutionary changes are either driven by recurrent consecutive fixations of beneficial novel genotypes (Arms Race dynamics) or by nonconstant selection maintaining the presence of multiple coexisting genotypes (Fluctuating Selection dynamics) (*6*–*9*). To quantify how selection varies through time and gain a more complete understanding of how these regimes affect genome-wide variation, temporally resolved information for both antagonists (*10*) is essential.

During coevolution, selection imposed by the antagonist varies depending on abundances of both interacting partners. Rapid evolution of traits with which species interact decreases the performance of antagonists and can, hence, affect population sizes (*11*). Because antagonist’s encounter rates depend on density, changes in population size alter the strength and sometimes the direction of selection (*12*), giving rise to a feedback between evolutionary and ecological change (*13*). Incorporating feedbacks in theoretical methods of coevolution suggests that genetic change is often more complex than simple oscillations in fluctuating selection regimes (*14*, *15*). Nonconstant population size and antagonistic selection were also suggested to affect molecular evolution by increasing the rate at which populations diverge and altering relative substitution rates of synonymous to nonsynonymous mutations (*16*). So far, we lack understanding of the buildup, maintenance, and purging of genetic diversity under rapid coevolution and eco-evolutionary feedbacks (*17*).

Time-resolved imaging of genomic change under constant selection shows that molecular evolution can be unexpectedly multifarious (*18*, *19*). In particular, in large (microbial) populations, de novo mutations generate variation rapidly enough for beneficial alleles to be common. This causes clonal interference of beneficial mutations within populations (*20*, *21*) and genetic hitchhiking, where selection acting on a mutation of large phenotypic effect influences the frequency trajectories of physically linked mutations with no or smaller effects (*22*, *23*). Both clonal interference and genetic hitchhiking are more common in the absence of recombination (*24*). Constant environmental selection over longer evolutionary time scale results in the frequent occurrence of adaptive mutations in the same genes, but their temporal changes in frequency vary considerably between replicates (*19*). All these suggest that genome architecture and size influence the dynamics of molecular change. Coevolving antagonists often differ in that respect (*10*), but empirical information on how this affects molecular dynamics is scarce.

Here, we assessed the effects of antagonistic coevolution and an eco-evolutionary feedback on temporal genomic change in a microbial host-virus system. We ran an experimental evolution study using initially isogenic algal host (*Chlorella variabilis* strain NC64A) and initially isogenic double-stranded DNA (dsDNA) virus (PBCV-1) in replicated chemostat systems (*n* = 3) (*16*, *25*). Both species reproduce exclusively asexually. The host reference genome is 46 Mb in size, with 74% non–protein-coding region (*26*), while the virus has an estimated genome size of 330 kb, with only 21% non–protein-coding (*27*). We tracked genomic, phenotypic, and population size changes of host and virus populations over time (100 days ~ 100 generations). Results from previous work with only the algal population that ran in parallel to the host-virus chemostats showed that host resistance does not evolve in the absence of the virus (*16*, *25*). Thus, all observed population size and phenotypic changes are likely the result of the species interaction. During coevolution, we anticipated dynamics of molecular change that are species specific due to differences in genome architecture and genome size of the coevolving antagonists. We also expected that the rapid population expansions of coevolving host and virus were driven by eco-evolutionary dynamics and predicted that they temporally match the dynamics of molecular change.

## RESULTS

### Population size and phenotypic dynamics translate into fluctuating fitness

**Fig. 1:**
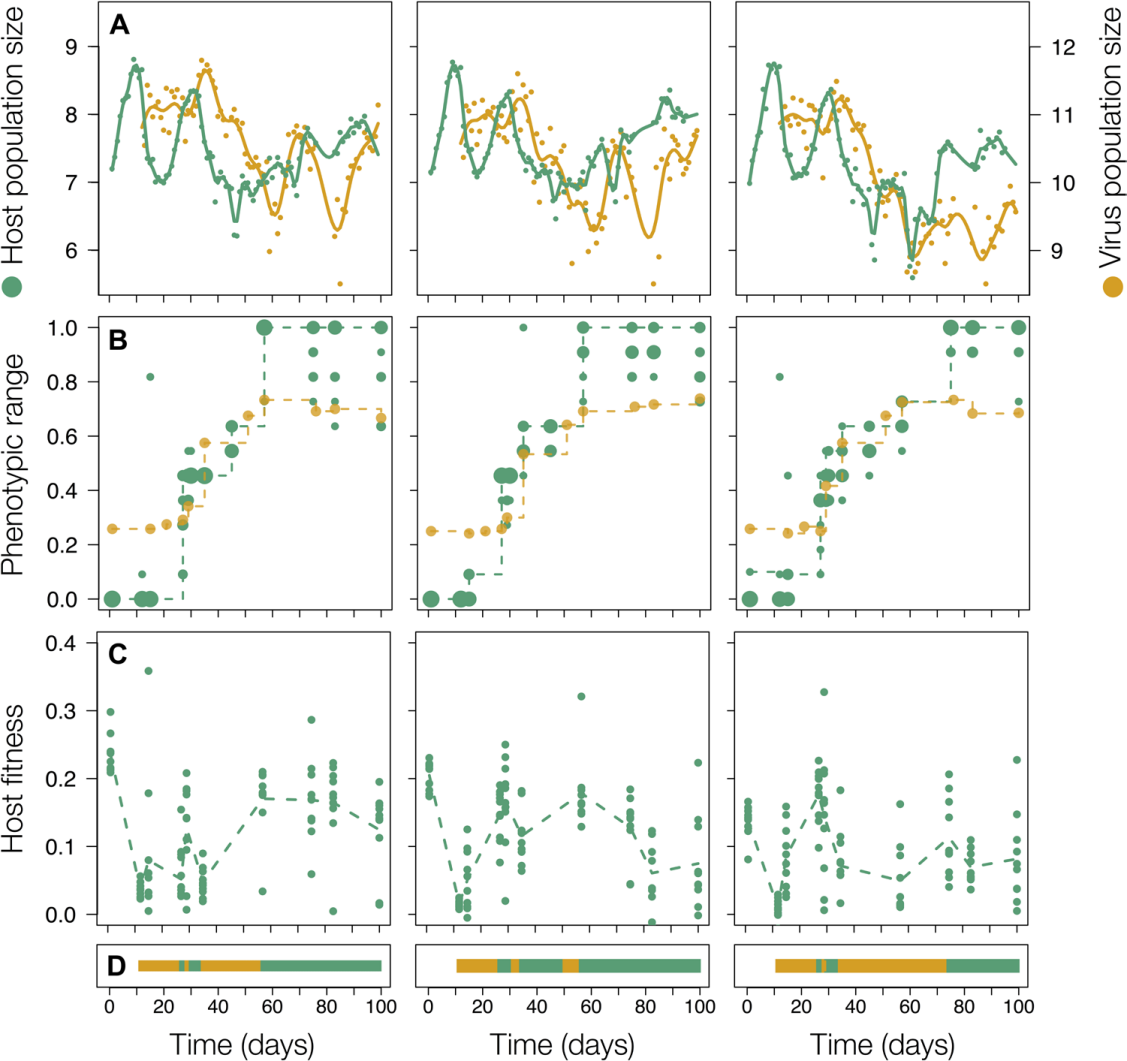
Ecological and evolutionary change of coevolving host and virus populations. The three columns correspond to three replicate experiments. (**A**) Population sizes of host (green, first vertical axis) and virus (orange, second vertical axis) were assessed daily (dots, total population size, log_10_ scale) and smoothed using cubic splines (lines). (**B**) Phenotypic evolution of resistance (green), i.e., the proportion of virus populations from all time points a host is resistant to, with circle size corresponding to the number of clones with the same phenotype and of infectivity (orange), i.e., the proportion of host individuals a virus population can infect. Dotted lines represent the maximum range per time point that was maintained at consecutive time points. Assays were done for 1320 comparisons of 10 host clones and one virus population collected from, respectively, 12 and 11 time points. (**C**) Fitness (growth rate per day in the presence of contemporary virus) of host individuals. (**D**) Binary visualization of the phenotypic interaction, with orange indicating a phenotypic match and green indicating a mismatch.

Host and virus population sizes initially showed damped cycling of two orders of magnitude (Fig. 1A). These dynamics were highly repeatable across replicates with a phase shift of 0.09 ± 0.4 days between the replicates (means ± SD). We observed three rounds of increases in host resistance range (i.e., proportion of virus populations a host clone is resistant to) and two rounds of increases in virus infectivity range (i.e., proportion of host clones a virus population is able to infect; Fig. 1B and fig. S1, full infection matrix). Host fitness (i.e., growth rate in the presence of contemporary virus) increased and decreased in concert with these phenotypic changes (Fig. 1C). Host and virus coevolved asymmetrically with the evolution of a generalist-resistant host (host individuals resistant to virus from every time point) at day 57 or 75. After this, population dynamics stabilized, and maintenance of variation in the host populations was driven by the cost of increasing resistance (fig. S2) (*25*). In summary, the antagonistic species interaction resulted in ecological dynamics that were temporally highly variable, including intervals of rapid population increases and decreases.

We identified changes in ecological interaction between the two antagonists by categorizing the time intervals as phenotypic match or phenotypic mismatch. We defined the phenotypic match and mismatch intervals based on time points when the host evolved resistance and the virus evolved infectivity. Phenotypic mismatch intervals started when hosts became more resistant and ended when the virus counteradapted to this resistance. Phenotypic match intervals started when the virus evolved higher infectivity and ended when the host counteradapted. We used the infection matrices (fig. S1) to identify time points when resistance and infectivity evolved. In intervals with a phenotypic match, selection for host resistance was strong, while we expect virus populations to increase in size. On the other hand, a phenotypic mismatch warrants pressure on the virus populations to evolve new means of infectivity, while it allows the host to grow without antagonistic restraints. Experiments started with a phenotypic match, and then match and mismatch alternated, followed by a phenotypic mismatch after the generalist has evolved.

### Rapid molecular change differs between both species

To follow evolutionary change on the genome level, we sequenced whole genomes of samples from 12 time points for each replicate to obtain population data (Pool-seq) for both species simultaneously. For analysis, we exclusively focused on single-nucleotide polymorphisms (SNPs) absent from the ancestral population, hereafter called derived alleles (table S1). To unambiguously distinguish variants from sequencing errors, we only report SNPs that reached appreciable frequencies at more than one time point. Because of differences in sequencing coverage between the species, variants never or only once reaching a frequency >0.05 in the virus or >0.25 in the host were excluded (see Materials and Methods for details on filtering procedure). Because the experiments were started with an isogenic culture grown from a single cell, we cannot exclude that some of the observed variants were already present at a frequency below our detection limit in the starting culture. It is, however, unlikely that low-frequency variants are particularly common in a freshly grown culture. Hence, the genomic change presented excludes various technical artifacts and rare and low-frequency variants but adequately represents the variation governed by selection and selective interferences.

**Fig. 2:**
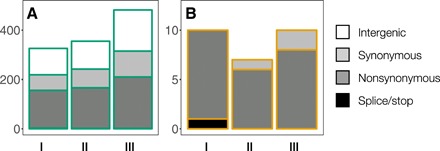
Molecular evolution differs between host and virus populations. (**A** and **B**) Overall number of observed genetic changes of host (A) and virus (B) populations colored by the predicted severity of their effect on protein structure (phenotype). Intergenic regions cover 74 and 21% of the host and virus reference genomes, respectively. Bars correspond to the three replicate experiments.

Dynamics of molecular change play out differently between the two species. The total number of derived alleles per replicate was 388 ± 83 in the host and 9 ± 2 (means ± SD, three replicates) in the virus populations, respectively. In the host populations, more than half of the observed mutations (211 ± 55) were synonymous or occurred in intergenic regions (Fig. 2A). The majority of these changes have no or minor phenotypic effects, suggesting that their substantial allele frequency changes are rather due to a physical association with mutations of larger selective advantage (genetic hitchhiking) (*22*). In contrast, the majority of the SNPs observed in the virus populations (20 of 21 unique SNPs) were nonsynonymous (Fig. 2B), meaning that they are likely candidates driving the coevolutionary dynamics rather than hitchhiking on an adaptive background. A small genome densely packed with protein-coding regions, skewed offspring distributions, and stronger purifying selection all contribute to the lack of genetic hitchhiking in viral evolution (*28*).

### Adaptation-induced host growth facilitates buildup of neutral genetic diversity

**Fig. 3:**
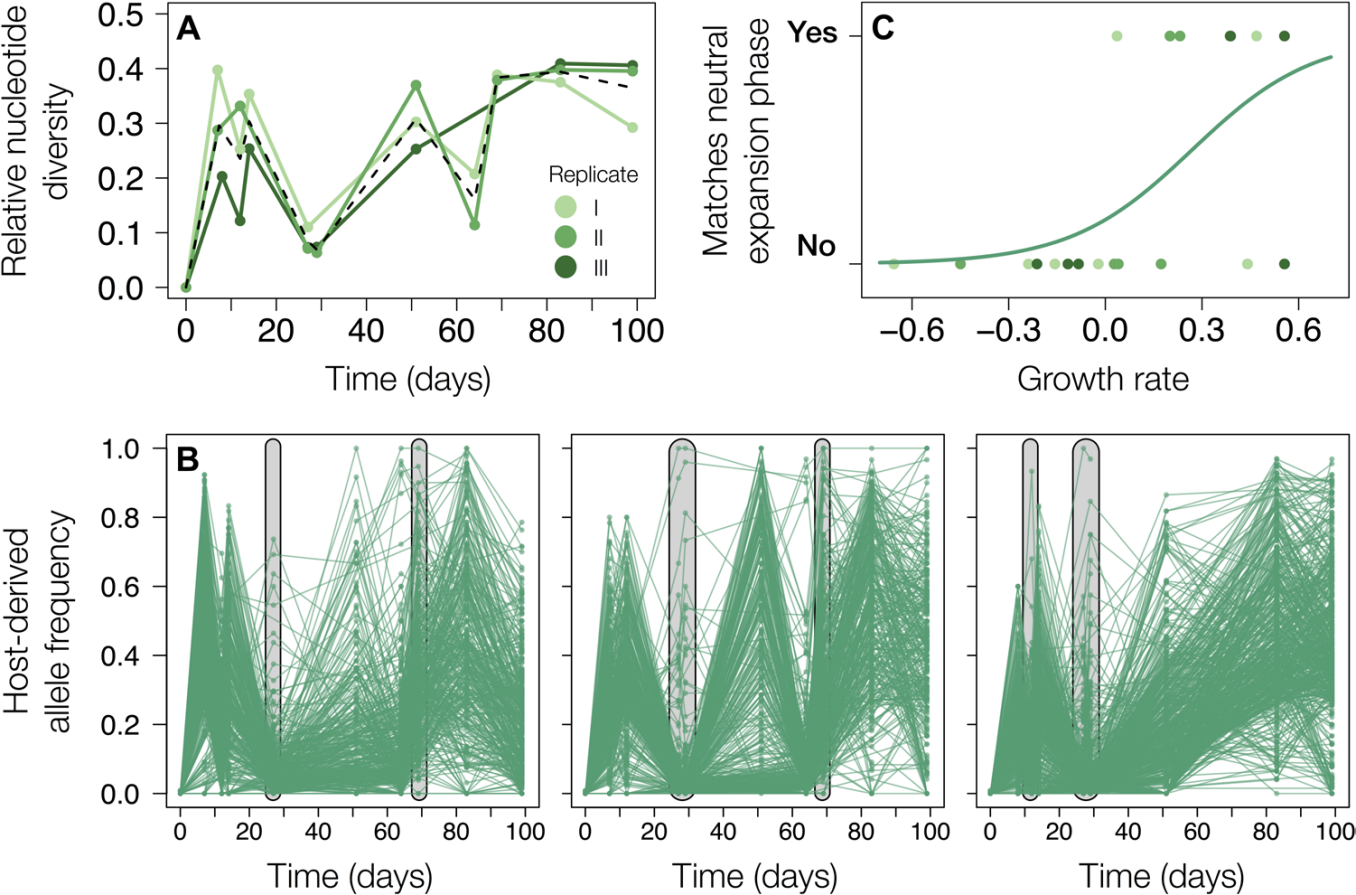
Adaptation and population expansion reciprocally influence each other. (**A**) Nucleotide diversity for the host populations was calculated per time point using derived allele frequencies of all loci that exhibited variation over the time course of the experiments. Lines are colored by replicate, with the black dashed line indicating the average. (**B**) Host allele frequency trajectories of all derived SNPs, with every line corresponding to an SNP. Distributions of VAFs match expectations under a neutral population expansion (Luria-Delbrück model) at the time points highlighted in gray. (**C**) Indicator variable reflecting if the distributions of VAFs in the host population matched the expectations of a neutral expansion phase (*y* axis) plotted against population growth in the 3 days leading up to the corresponding time point. Every dot reflects a time point and is colored by replicate, and the line corresponds to a generalized linear model fit with replicate as random effect.

Our temporal resolution allowed us to detect the signature of a selective sweep in the host populations at two time points. In every replicate, within-population diversity (Fig. 3A) markedly dropped in the time intervals preceding days 27 and 64. Such a diversity reduction is characteristic for a selective sweep when a low-frequency beneficial mutation quickly rises to fixation and physically linked variants are dragged along, eliminating genetic variation (*29*). Host selective sweeps coincided with—and likely caused—increases in resistance range and host population size (Figs. 1 and 3).

For each time point, we compared the distribution of variant allele frequencies (VAFs) in the host to a neutral null model. As such, the classic Luria-Delbrück model predicts that the distribution of VAFs can be described by a simple power law distribution, assuming a neutral population expansion (*30*–*32*). Any violation of those assumptions, e.g., the absence of population growth or the presence of multiple coexisting cohorts governed by clonal interference, is likely to cause a deviation from such a distribution. At time points showing such a power law fit, neutral processes are sufficient to explain the distribution of VAFs. Therefore, we interpret this as evidence that the population consists of one expanding cohort of equally fit cells. Such dependencies can be observed when neutral de novo mutations occur at a reasonably high rate, which is feasible for *Chlorella* (see Materials and Methods for more details).

The distribution of VAFs in the host revealed a power law dependence at the same time points when genome-wide diversity was reduced (Fig. 3B and fig. S3; additional time point in replicate III at day 12). This indicates that at the time points where we identified sweeps, the populations consisted of one cohort all carrying an acquired highly beneficial mutation, and the observed genetic variation (within this cohort) is shaped primarily by neutral evolutionary processes. Regressing the presence of this dependence on host population growth rate in the 3 days leading up to the corresponding time point revealed a positive correlation (generalized mixed-effect model with family binomial and replicate as random effect: *z* = 2.175, *P* = 0.0297; Fig. 3C). This is consistent with our prediction that sweeps (and matches to the Luria-Delbrück model) coincided with host expansion phases. In summary, only directly after a sweep did host populations consist of one expanding cohort, and the observed rapid (re)generation of genetic variation is facilitated by the expansion.

### First viral genetic change coincides with population growth

**Fig. 4:**
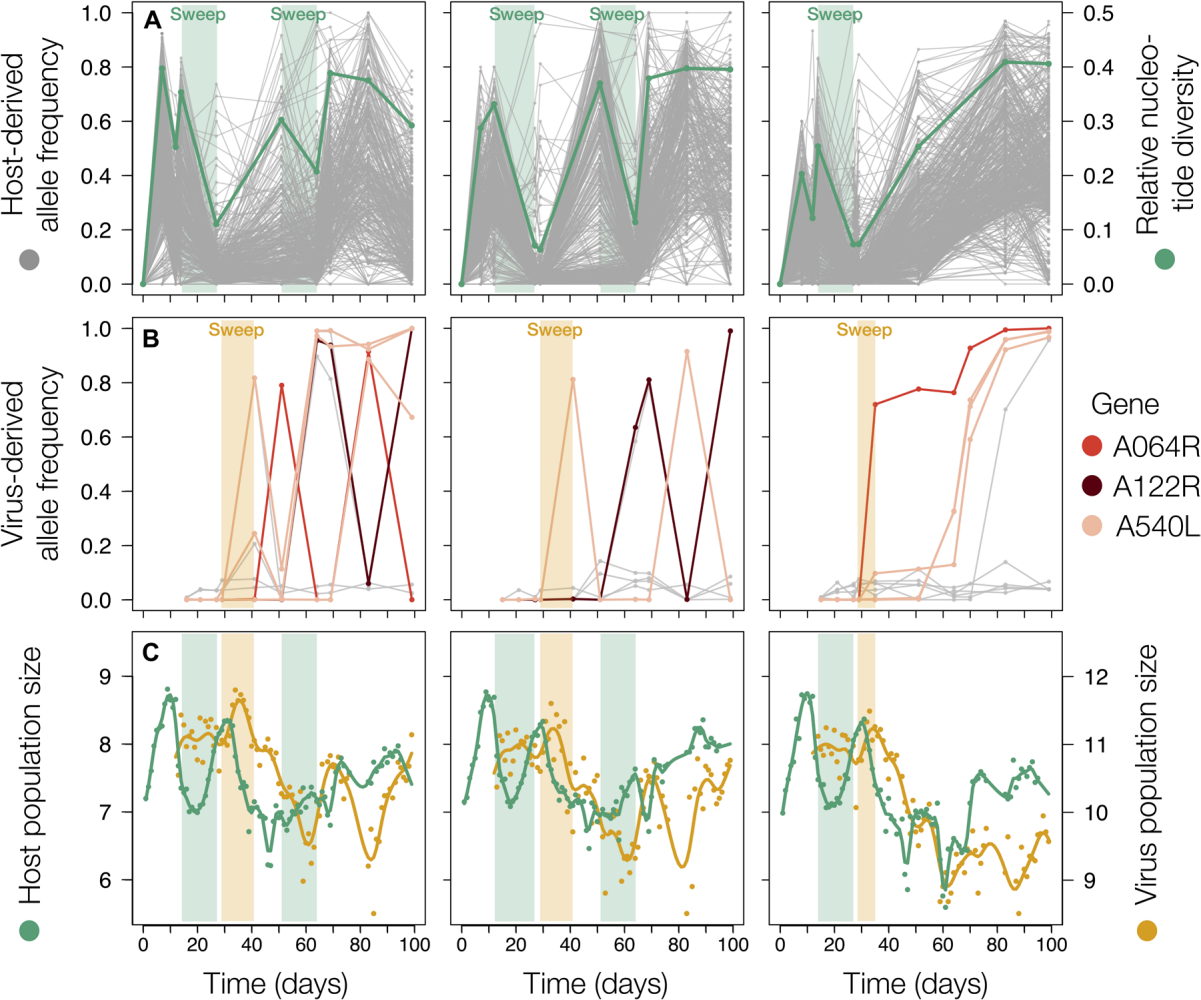
Repeatable selective sweeps match periods of population expansions in both populations. (**A**) Allele frequency trajectories of all derived SNPs are plotted for the host in gray (first vertical axis). Sweeps are evidential by a decrease in relative nucleotide diversity (second vertical axis), plotted in green. Light green highlight indicates the host sweeps identified. (**B**) Derived allele frequency trajectories for the virus. Gray lines reflect trajectories of mutations observed in one replicate only. Colored lines indicate trajectories of variants that were observed in more than one replicate, colored by the gene they occur in. Light orange highlight indicates the first virus sweep that occurs at the same time in all three replicates. (**C**) Population sizes of host (green) and virus (orange) were assessed daily (dots, total population size, log_10_ scale) and smoothed using cubic splines (lines). Light green and orange shadings indicate periods that match with sweeps as indicated in (A) and (B).

A first sweep in the virus populations occurred directly after the first host sweep between days 29 and 41. After none of the derived variants showed noticeable frequency changes across the first three time points sampled, a previously undetected variant increased in frequency over 70% in this time interval in every replicate (Fig. 4B). This first sweeping allele did not monotonically increase to fixation in any of the replicates; however, such a marked frequency change (>70% in ~12 generations) implies a large selection coefficients (0.90 ± 0.35, means ± SD across three replicates). The ecological impacts of this first virus sweep were virus population growth, host decline, and the interaction reverted to a phenotypic match (Figs. 1D and 4, B and C). After evolution of a generalist host, the ecological interaction remained a mismatch because the majority of the host cells were resistant to the contemporary virus population (fig. S1). Derived viral alleles both substantially increased and decreased in frequency, and patterns of genomic change were less consistent between replicates than the ecological dynamics.

### Repeated frequency increase suggests positive selection

In the host populations, 294 of a total of 703 SNPs were found multiple times and 166 occurred in all three replicates (fig. S4). The probability of observing the same mutation more than once by chance is small (table S2), hence suggestive of the action of natural selection. More than half of the repeated mutations (219 ± 35) were already observed before the introduction of the virus at day 12, and they might provide a benefit in the abiotic environment or during competitive growth rather than confer resistance. We expect variants in genes affecting resistance to increase in frequency at time intervals leading up to those time points where sweeps were detected, and we evaluated which SNPs fit this criterion. Potential candidate genes for the evolution of resistance are genes affecting outer cell wall composition, alterations of which would prevent viral recognition of the host cell. However, we found no primary candidate genes among the high-frequency and repeatedly found SNPs at days 27 and 64 (tables S3 and S4). This could be because resistance was encoded by a larger structural variant (*16*), which we do not evaluate here. In addition, cell walls and the extracellular matrix are complex biological structures regulated by many genes, and it is quite conceivable that different mutations have similar phenotypic outcomes or that the evolution of resistance is mediated by multiple mutations of moderate effect.

In the virus populations, 5 of 21 unique mutations were observed in more than one replicate (fig. S4). All five were nonsynonymous, and three of them—among which the one mutation observed in every replicate—altered the protein structure of the gene *A540L*, which is highly conserved within the 41 chlorovirus isolates sequenced to date (*33*). A fourth mutation was in the gene *A122R*. Both these genes have no known orthologs outside the family of chloroviruses and are expressed during the late-infection stage, i.e., after viral DNA synthesis has begun (*27*). The fifth mutation was in the first glycosyltransferase domain of the gene *A064R*, encoding for a large protein present in every chlorovirus sequenced so far (*33*). The protein encoded for by *A064R* plays an important role in the construction of the major capsid glycoprotein that makes up the outer virus particle; PBCV-1 mutants that have a truncated version of the gene are viable but less stable (*34*). The functional annotations and high degree of conservation of these three repeatedly mutated genes support the notion that the mutations we detect in the virus were driving the observed dynamics rather than the genetic hitchhikers.

## DISCUSSION

Adaptation by natural selection is based on the premise that genetic variation, which enhances reproductive success, increases in frequency over generations. Here, we have documented frequency change on a fine temporal scale, in an experimental system comprising two species that are each other’s primary selective agent. Recent experimental studies on molecular evolution in host-virus systems showed that coevolution accelerates molecular evolution (*35*), viral diversity increases rates of host adaptation and diversification (*3*), and coevolutionary selection can produce different signatures in coevolving species (*10*). We here provided temporally resolved information on population size, phenotypic evolution, and genomic change for both interacting partners. We identified adaptive sweeps with temporal consistency in both species, showed molecular evolutionary differences between the species, and empirically demonstrated the feedback between ecological and evolutionary change across multiple levels of biological organization.

The genome-wide patterns of molecular evolution differed markedly between the species, illustrated by the different total number of observed mutations (Figs. 2 and 4, A and B). Viral evolution was confined to mutations in three candidate genes for infectivity evolution, which are all specific to and highly conserved among chloroviruses. Hence, architectural properties of virus genomes and viral specific reproduction modes result in dynamics of molecular change dominated by a few potentially large effect mutations (*28*). In contrast, genetic diversity was higher and rapidly generated in the host populations, which allowed us to identify adaptive sweeps by the loss of genetic diversity (*29*). Both the overall number and the higher proportion of intergenic and synonymous SNPs (Fig. 2) suggest that genetic hitchhiking is pervasive in the host populations. This can be explained by the large population sizes reached in our chemostats, the lack of recombination, and the strong selection imposed by the virus (*18*, *22*, *23*, *36*). In summary, differences in genome architecture and reproduction modes resulted in distinctly different dynamics of molecular evolution between the coevolving species.

We found evidence for an eco-evolutionary feedback in the host by linking genetic diversity changes to population size dynamics. While most previous host-virus coevolution experiments were done with constant population sizes or serial transfers to new medium [e.g., (*3*, *10*, *35*)], our chemostat setup allowed the coevolutionary interaction to unfold without interference with population sizes (Fig. 1). This enabled us to investigate the dynamic influence of adaptation on population size and vice versa. Adaptive sweeps coincided with increases in resistance and population growth (Fig. 4). In the host, neutral processes are sufficient to describe the observed VAF distribution at these time points (Fig. 3B and fig. S3) (*30*). This suggests that the interaction with the virus frees the algal clonal lineage that acquires a resistance mutation from selection. While this lineage expands, neutral mutations accumulate with each cell division (*37*), and genetic diversity increases at a time scale comparable to how it is purged during sweeps. We were not able to link this genetic diversity to functional traits, but the fact that genetic diversity is generated so rapidly likely has implications for adaptation to multidimensional (natural) environments. In summary, evolution was both a cause (increased resistance) and a consequence (neutral diversity buildup) of ecological change (population expansion).

Our results demonstrate how both interspecific differences in substitution supply rate between the species and eco-evolutionary feedback dynamics influence molecular change during host-pathogen coevolution. We anticipate that a more widespread recognition of the various ways by which ecological and evolutionary change can affect each other will be essential to interpret the genomic signature of evolution under species interactions and understand the mode, pace, and predictability of evolution in natural communities.

## MATERIALS AND METHODS

### Chemostat experiments

Experiments were performed in continuous flow-through systems (chemostats) as previously described (*25*) but using 1000 ml glass bottles containing 800 ml of modified BBM (Bold’s basal medium with nitrate being replaced by equal moles of ammonium chloride). Previous experiments were conducted in 400 ml of medium; we increased the volume this time to ensure that sufficient samples for DNA collection (40 ml) could be taken without affecting the ecological and evolutionary dynamics. Sterile medium was continuously supplied to the chemostats at a rate of 0.1 per day. Chemostats were continuously mixed by stirring and maintained at 20°C under light conditions. Six chemostats were inoculated from the same liquid culture derived from a single algal clone so that all algal host populations started from the same ancestor. Isogenic virus was added to three chemostats at day 12. Three replicates without virus served as controls to demonstrate that host populations grew to carrying capacity and resistance did not evolve (fig. S5).

Algal and virus densities were assessed daily. Algal samples were fixated with 2.5% Lugol for later algal quantification using imaging flow cytometry (FlowCam, Fluid Imaging Technologies Inc.). For virus enumeration, samples were fixated with 1% glutaraldehyde, flash frozen in liquid nitrogen, and stored at −80°C. Virus particles were counted using flow cytometry (FACSCalibur, Becton Dickinson, San Jose, California) following (*38*) and (*25*). In regular intervals, algal and virus subpopulations were preserved for subsequent phenotyping [see (*25*) for details]. Simultaneously, 40 ml was taken from each chemostat, concentrated using ultracentrifugation at ~35,000*g* for 2 hours, and the pellet was frozen at −80°C for DNA extractions.

Log-transformed population sizes of both species were smoothed using cubic splines [spline function in R (*39*)]. We tested parallel changes in host population sizes across replicates using wavelet coherence analysis as previously described (*16*). Briefly, we measured the local correlation between two time series and identified the dominant and significant phase shifts between them (*40*). The value of wavelet coherence is “0” when there is no relation between the two oscillations (no phase coupling) and “1” when there is a full correlation (perfect phase coupling) between the two oscillators. We extracted from these analyses the significant phase shifts (α < 0.05 and outside the cone of influence). Statistical significance of the empirical correlation between replicates was assessed by testing the null hypothesis of absence of correlation with the WaveletComp R package, which allows for simulation of white noise (default methods) (*40*). We restricted analysis to the time period from day 0 to day 55, as algal populations stabilized after this.

### Phenotypic assays

To follow the coevolution of resistance and infectivity of host and virus, 11 time points were selected on the basis of population size dynamics. For each time point, 10 algal clones were randomly isolated from previously plated populations on agar plates and regrown in BBM. Afterward, each host clone was exposed to virus populations from all time points. To determine the resistance or susceptibility of the algal clones toward the virus populations, their growth when exposed to virus (initial ratio of algal cells to virus particles: 0.01) was compared with the growth of the algal clone in the absence of the virus. Growth rates were calculated on the basis of optical density measurements (Tecan, Infinite M200PRO, 680 Männedorf, Switzerland) measured at time point 0 and after 72 hours. A host clone was deemed susceptible when the mean growth rate plus 2 SDs of four technical replicates in the presence of the virus was lower than mean growth rate minus 2 SDs of the control or when mean growth rate in the presence of the virus was negative. Resistance and infectivity ranges were calculated as the proportion of virus populations a host individual was resistant to and the proportion of host individuals a virus population could infect, across sampled time points. Growth rates of algal clones measured in the absence of virus were correlated to resistance range to estimate trade-offs between resistance and growth rates.

We defined phenotypic match and mismatch intervals based on resistance and infectivity evolution over time. Resistance evolution occurred when hosts from a given time point became resistant against virus from their past and contemporary time points (horizontal arrows in fig. S1). Infectivity evolution occurred when virus became able to infect previously resistant hosts, i.e., when contemporary virus or virus from the next time point could infect hosts, whereas the same hosts could not be infected by virus from past time points (vertical arrows in fig. S1). As we do not have continuous data, we marked these transitions between the different stages by arrows in the infection matrix. Note that we only took observations into account when the phenotype was maintained in the next time step. Because we were only interested in identifying when resistance and infectivity evolved, we marked transitions at the time point when a phenotype was first observed and did not take into account the frequency at this time point.

### Sequencing design

DNA was extracted from frozen and concentrated samples using the DNeasy Blood and Tissue Kit (Qiagen) with minor modifications. First, 100 μl of buffer ATL + 30 μl of Proteinase K were added to 200 μl of concentrated sample, followed by an incubation at 56°C for 4 hours, continued by adding 600 μl of 1:1 buffer AL + ethanol mix and proceeding by following the standard column-based protocol. Paired-end libraries were prepared using a Nextera XT library preparation kit. Twelve time points from one replicate (replicate 3) were sequenced in four runs of an Illumina NextSeq (Illumina NextSeq 500 high output) machine. Twelve time points from the other two replicates of coevolution were first sequenced in five runs. The amount of DNA obtained for host and virus was correlated to their relative population sizes, which, for some time points, led to comparatively low genome-wide coverage for the host. Six more NextSeq runs were performed to mitigate this issue and increase coverage for selected time points where we anticipated low host coverage.

### Read processing

Quality of the reads was assessed using FastQC. For some runs, for which base calling qualities dropped notably toward the end of the reads, read tails were trimmed using Trimmomatic v0.35 (*41*) with default settings. We ran SeqPrep v1 (https://github.com/jstjohn/SeqPrep) to merge read pairs in case forward and reverse reads overlapped and subsequently remove reads shorter than 70 base pairs (bp). Reads were mapped with bwa mem v0.7.12 with slightly increased accuracy setting (-r 1) (*42*) to one reference genome consisting of both host (*26*) and virus (*27*) reference sequences. We ran Picard (v2.0.1) FixMateInformation and AddOrReplaceReadGroups (http://broadinstitute.github.io/picard), removed alignments with quality 0 with awk, and merged data per time point (across lanes and sequencing runs) using SAMtools v1.3 (*43*). Resulting .bam files containing aligned reads per time point were sorted, cleaned, and indexed with Picard SortSam, Picard CleanSam, and SAMtools index. For the virus, average genome-wide coverage was >1000× at all time points where virus was present. We used the SAMtools view with the -s parameter to downsample to an average per-position coverage of 1000×; this makes the subsequent analysis less computationally demanding but keeps the empirical coverage distribution intact for later filtering. Table S1 summarizes average coverage of host reads after quality control and alignment for every time point per replicate. If average per-position coverage was below 10×, the time point was completely removed from analysis. Coverage for specific sites was evaluated after .bam files were transformed to .sync format with SAMtools mpileup and Popoolation2 (*44*) v1.201 mpileup2sync.pl (--min-qual 1). In these .sync files, sites with a coverage outside of the interval (mean ± 3 SDs) or smaller or equal to 10× were set to “not available” with a custom R script. The frequency of the nonreference allele with the highest average frequency across time points was calculated as derived allele frequency.

### Variant calling and locus filtering

We applied several criteria to (remove sequencing errors, reduce technical artifacts, and) obtain only those alleles with patterns driven by selection, following other genomic time series analyses (*19*, *23*, *24*) unless there was reason to deviate. It is inherent to pooled sequencing data that low-frequency mutations are difficult to distinguish from sequencing errors (*45*). To remove any observed variation induced by sequencing error, we set a conservative coverage-based detection limit threshold of 5% (virus) and 25% (algae). For any indel calls passing this threshold, all information 10 bp up- and downstream (including the position itself) was not included for further analysis. Because we started the experiments with isogenic populations, any observed variation at time point 0 (<99% ancestral allele frequency) is likely an artifact, and these loci were removed from the dataset. We only included a locus if derived allele frequency reached the detection threshold at more than one time point. Loci were also removed if the number of missing values across all time points exceeded 1 (virus) or 3 (host). Last, in the host datasets, we observed several sets of mutations at closely neighboring reference positions with highly correlated frequency trajectories. Our setup did not permit us to ascertain if those are multiple independent polymorphisms in the same cohort or a larger structural variant that appears as multiple SNPs in our alignment. Hence, SNPs within 1000 bp of each other and with highly correlated frequency trajectories were collapsed into one. Frequency values were averaged, and during annotation, the most severe phenotypic effect was used as representative for these collapsed sets. Because we expect selection to be nonconstant under the described dynamic eco-evolutionary conditions, we did not filter allele frequency trajectories for lack of temporal autocorrelation. Table S2 shows the number of allele frequency trajectories removed by each filtering step. The final set of SNPs was annotated using snpeff (*46*).

Given the observed population sizes and length of the experiment, it is highly unlikely for spontaneously arising neutrally evolving loci to reach the detection limit by drift alone. Any allele that reaches a detectable frequency is therefore either under positive selection or linked to something that is. We acknowledge that because we rather stringently filtered for potential sequencing errors, we inevitably also excluded low-frequency genomic variants from the analysis and thus did not exhaustively characterize the genomic variation in the populations. However, this temporal genome-wide SNP dataset does provide an accurate image of the strength and speed of evolution.

### Genetic diversity in an expanding population

In an exponentially growing population, mutations can rapidly increase in frequency. If all individuals (cells) have comparable growth rates (i.e., when apparent fitness differences are absent), the expected distribution of VAFs generated by the underlying stochastic process of de novo mutation follows a Landau distribution (*31*), first detected in the famous Luria-Delbrück experiment (*37*). We showed before (*30*) that the VAF distribution of a neutrally exponentially growing population should follow a power law given by a cumulative distribution

$$M(f)=\frac{\mu }{\beta }(\frac{1}{f}-\frac{1}{f_{\text{max}}})$$

Here, *f* denotes the frequency of a variant within the population, μ is the mutation rate, and β is an effective rate of surviving offspring. We then defined the universal neutrality curve $\bar{M}(f)$ given an appropriate normalization (*32*). The curve is defined as

$$\bar{M}(f)=\frac{\frac{\mu }{\beta }(\frac{1}{f}-\frac{1}{f_{\text{max}}})}{\text{max}(M(f))}$$

If one plots this against $\frac{1}{f}$, then the universal neutrality curve becomes a linear line in the interval [0,1] that is independent of the mutation and offspring survival rates. In other words, if variants were generated by random de novo mutations during an expansion phase without fitness differences between individuals, genetic diversity collapses onto this line. We plotted all variants in the frequency interval between 5 and 40% and used the area under the curve (AUC) and the Kolmogorov distance from the line *y* = *x* to assess goodness of fit of a linear regression. Any time point with a Kolmogorov distance lower than 0.25 and with an AUC below 3 was judged as a good fit. Good fits to the linear regression indicate agreement between the theoretical scenario of an effectively neutral exponentially growing population and the empirically observed VAF distribution.

Considering a selective sweep, when a single highly adaptive cell grows exponentially, then to reach a frequency of 10% by hitchhiking alone, any neutral mutation needs to occur during the first 10 cell divisions this cell undergoes. This means that with a genome size of ~42 Mb, observing power law dependencies and rapid neutral allele frequency increases of 10% during sweeps are feasible if the *Chlorella* mutation probability is roughly 1/(42 × 10^6^ × 10) *=* 2.4 × 10^−9^ substitutions/position/cell division. While there are no good estimates of the mutation rate in *Chlorella*, such a rate is plausible. As the virus genome is orders of magnitude smaller (~330 kb), observing comparable power law dependencies would require a mutation rate of 1/(330 × 10^3^ × 10) = 3.0 × 10^−7^ substitutions/position/cell division. Although such rates have been observed for DNA viruses, no increases in genetic diversity after sweeps were observed. We attribute this to the fact that small virus genomes are densely packed with protein-coding regions; hence, neutral positions are rare. Because of the low number of variants, we did not attempt to do statistics on the viral VAF distribution.

We performed a logistic regression of time points where the VAF distribution matches the expectation under neutral expansion (largely, these are the same time points identified as sweeps due to reduced genetic diversity) on host population growth (generalized linear model with random effect, family = binomial) using the R package lme4 (*47*). We only included days when virus was present and averaged growth over the 3 days leading up to the time point of genetic sampling. Replicate was included as random effect to account for within-replicate dependency of data points.

### Probability of observing the same SNP in multiple replicates

Assuming that mutations evolve de novo, are neutral, and are uniformly distributed across the genome, every genomic position has the same probability of acquiring a mutation. Under these assumptions, observing a mutation in our experiments is equivalent to randomly drawing marbles from a vase without replacement. The size of the vase is equivalent to the number of genomic positions we evaluated with our sequencing approach, and the sample size (number of marbles drawn) equals the number of mutations observed per replicate. To assess how likely the empirically observed overlap is, we simulated such a sampling procedure using R and found that the probability of the observed overlap (fig. S4, A and B) is extremely small (*P* < 10^−5^). Because of, i.a., random variation in sequencing coverage and complex genomic regions, not every position of the reference genome can be reliably evaluated; hence, reference genome size is an overestimate of vase size. To get an idea of how large the pool of mutable sites need to be to reproduce the empirically observed overlap, we repeated the simulations for a grid of vase sizes using the empirical number of observed mutations (minimum across three replicates) as the total number of marbles drawn and reported the number of marbles drawn more than once (i.e., SNPs observed in multiple replicates) in table S2.

## Supplementary Material

http://advances.sciencemag.org/cgi/content/full/5/10/eaax0530/DC1

Download PDF

Data file S1

Data file S2

Data file S3

The feedback between selection and demography shapes genomic diversity during coevolution

## SUPPLEMENTARY MATERIALS

Supplementary material for this article is available at http://advances.sciencemag.org/cgi/content/full/5/10/eaax0530/DC1 Fig. S1. Full infection matrix highlights coevolutionary phenotypic changes. Fig. S2. Resistance is costly. Fig. S3. Genetic diversity after selective sweeps matches expectations under neutrality. Fig. S4. Repeatable genomic change provides evidence for the action of natural selection. Fig. S5. Ecological change is less dynamic in the absence of species interactions. Table S1. Sequencing and filtering statistics indicate the reliability of the genomic datasets. Table S2. Observing mutations in multiple replicates independently is unlikely under neutrality. Table S3. Functional annotations of SNPs at high frequency in host populations after selective sweep at day 27. Table S4. Functional annotations of SNPs at high frequency in host populations after selective sweep at day 64. Data file S1. Population sizes (observed and smoothed values). Data file S2. Results of phenotypic assays. Data file S3. Filtered derived allele frequencies.
