# Supplementary material for: The feedback between selection and demography shapes genomic diversity during coevolution
Source: Sci Adv. 2019 Oct 2;5(10):eaax0530. doi: 10.1126/sciadv.aax0530 (PMC6774728; doi:10.1126/sciadv.aax0530)
Supplement: http://advances.sciencemag.org/cgi/content/full/5/10/eaax0530/DC1 [file supp_5_10_eaax0530__index.html]

Science Advances | Science AdvancesAAASSearchScience AdvancesMenu

## Supplementary Materials

**The PDF file includes:**

- Fig. S1. Full infection matrix highlights coevolutionary phenotypic changes.
- Fig. S2. Resistance is costly.
- Fig. S3. Genetic diversity after selective sweeps matches expectations under neutrality.
- Fig. S4. Repeatable genomic change provides evidence for the action of natural selection.
- Fig. S5. Ecological change is less dynamic in the absence of species interactions.
- Table S1. Sequencing and filtering statistics indicate the reliability of the genomic datasets.
- Table S2. Observing mutations in multiple replicates independently is unlikely under neutrality.
- Table S3. Functional annotations of SNPs at high frequency in host populations after selective sweep at day 27.
- Table S4. Functional annotations of SNPs at high frequency in host populations after selective sweep at day 64.
- Legends for data files S1 to S3

Download PDF

**Other Supplementary Material for this manuscript includes the following:**

- Data file S1 (.csv format). Population sizes (observed and smoothed values).
- Data file S2 (.csv format). Results of phenotypic assays.
- Data file S3 (.csv format). Filtered derived allele frequencies.

**Files in this Data Supplement:**

- Adobe PDF - aax0530\_SM.pdf
