# Supplementary material for: The feedback between selection and demography shapes genomic diversity during coevolution
Source: Sci Adv. 2019 Oct 2;5(10):eaax0530. doi: 10.1126/sciadv.aax0530 (PMC6774728; doi:10.1126/sciadv.aax0530)
Supplement: Download PDF [file aax0530_SM.pdf]

Science Advances
AAAS

advances.sciencemag.org/cgi/content/full/5/10/eaax0530/DC1

Supplementary Materials for

The feedback between selection and demography shapes genomic diversity during coevolution

Cas Retel, Vienna Kowallik, Weini Huang, Benjamin Werner, Sven Künzel, Lutz Becks, Philine G. D. Feulner*

*Corresponding author. Email: philine.feulner@eawag.ch

Published 2 October 2019, *Sci. Adv.* **5**, eaax0530 (2019)
DOI: 10.1126/sciadv.aax0530

The PDF file includes:

Fig. S1. Full infection matrix highlights coevolutionary phenotypic changes.
Fig. S2. Resistance is costly.
Fig. S3. Genetic diversity after selective sweeps matches expectations under neutrality.
Fig. S4. Repeatable genomic change provides evidence for the action of natural selection.
Fig. S5. Ecological change is less dynamic in the absence of species interactions.
Table S1. Sequencing and filtering statistics indicate the reliability of the genomic datasets.
Table S2. Observing mutations in multiple replicates independently is unlikely under neutrality.
Table S3. Functional annotations of SNPs at high frequency in host populations after selective sweep at day 27.
Table S4. Functional annotations of SNPs at high frequency in host populations after selective sweep at day 64.
Legends for data files S1 to S3

Other Supplementary Material for this manuscript includes the following:

(available at advances.sciencemag.org/cgi/content/full/5/10/eaax0530/DC1)

Data file S1 (.csv format). Population sizes (observed and smoothed values).
Data file S2 (.csv format). Results of phenotypic assays.
Data file S3 (.csv format). Filtered derived allele frequencies.

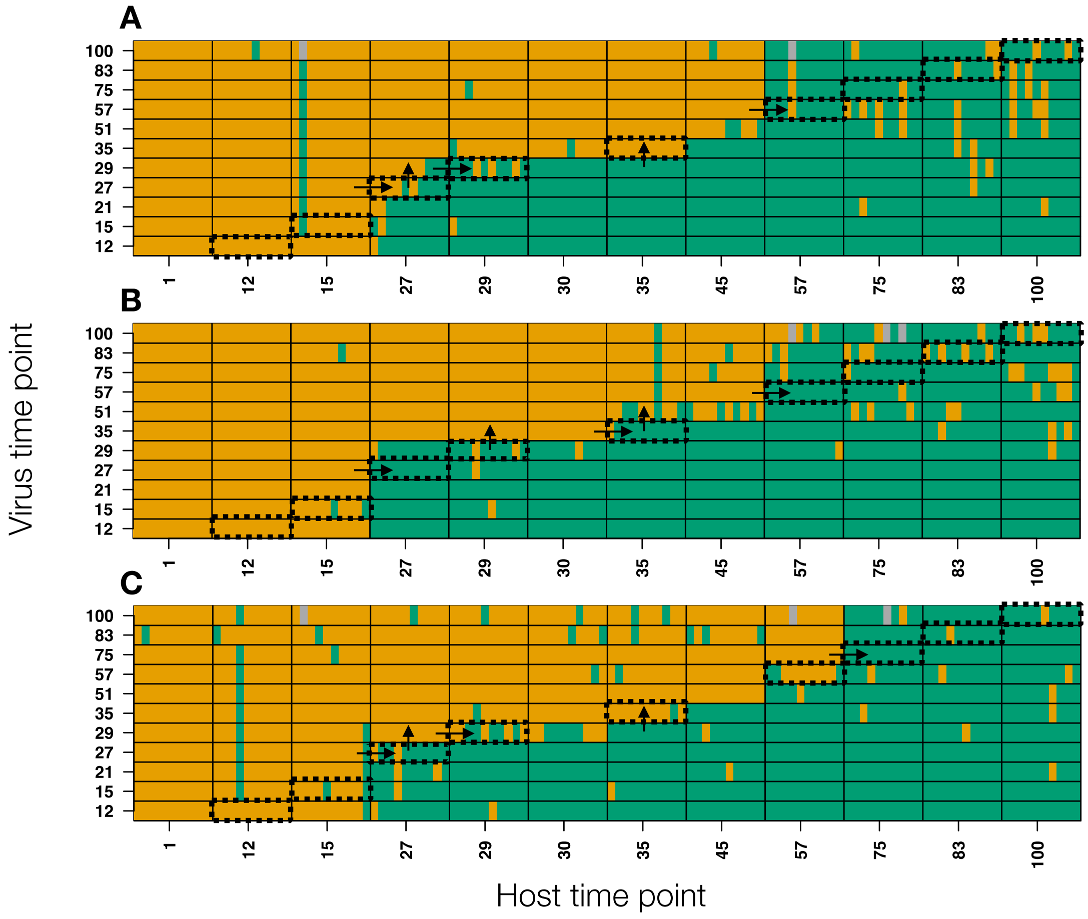

Fig. S1. Full infection matrix highlights coevolutionary phenotypic changes. For each pairwise combination of time points (black outlined rectangles) ten host clones (horizontal axis) and one virus population (vertical axis) were tested in comparative growth assays. Green squares (inside each of the boxed rectangles) indicate that the host clone was resistant, orange squares indicate host was susceptible to infection by the virus. Grey squares indicate missing data. Contemporary comparisons are highlighted by thicker dotted boxes. Horizontal arrows indicate that algae evolved from being susceptible at the time point before to being resistant (a change from match to mismatch). Vertical arrows indicate virus evolution; from not able to infect host from past time points to be able to infect host (a change from mismatch to match). Figures **A**, **B** and **C** correspond to replicates I, II and III, respectively.

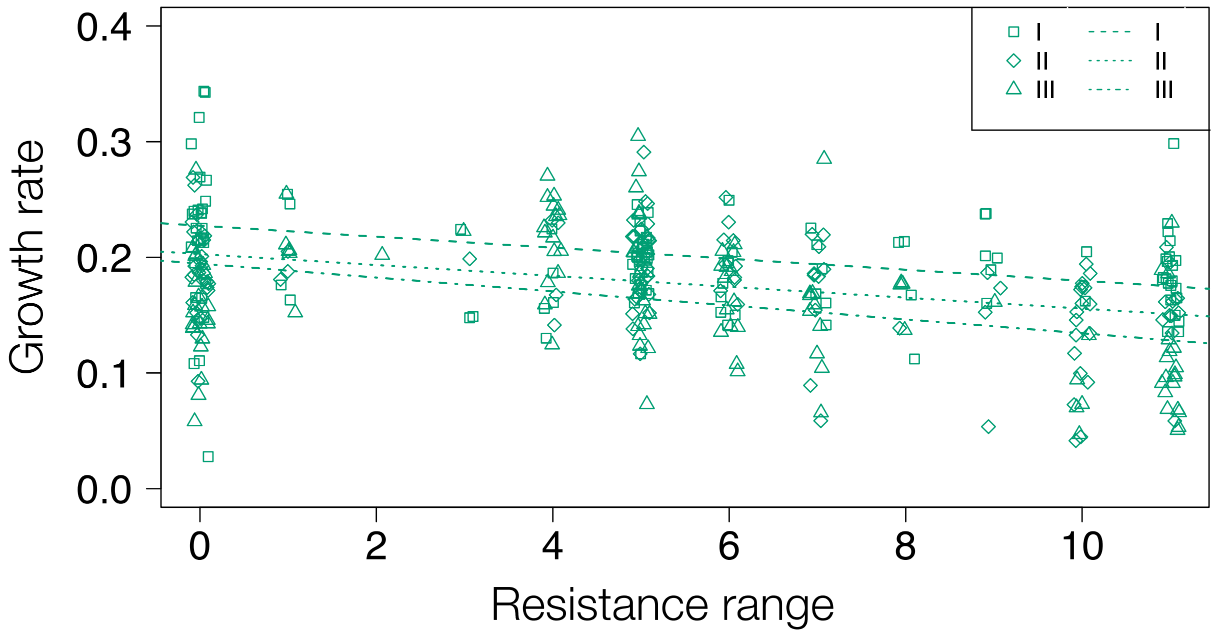

Fig. S2. Resistance is costly. For each host population, host growth rate in absence of virus is plotted against resistance range (0 corresponding to susceptibility to virus from every time point, 11 indicating resistance against every virus). Linear regressions of growth rate on resistance range yields negative slope estimates of $5.2*10^{-3} \pm 7.4*10^{-4}$ (p-values always smaller than 0.01) for the three respective replicates; fits are shown as dotted lines. The negative correlation indicates the presence of a tradeoff between resistance range and growth in absence of the virus, which allows maintenance of multiple resistance types in the host population after the generalist evolves (*28*). The legend shows symbols and line types used for the three replicate experiments.

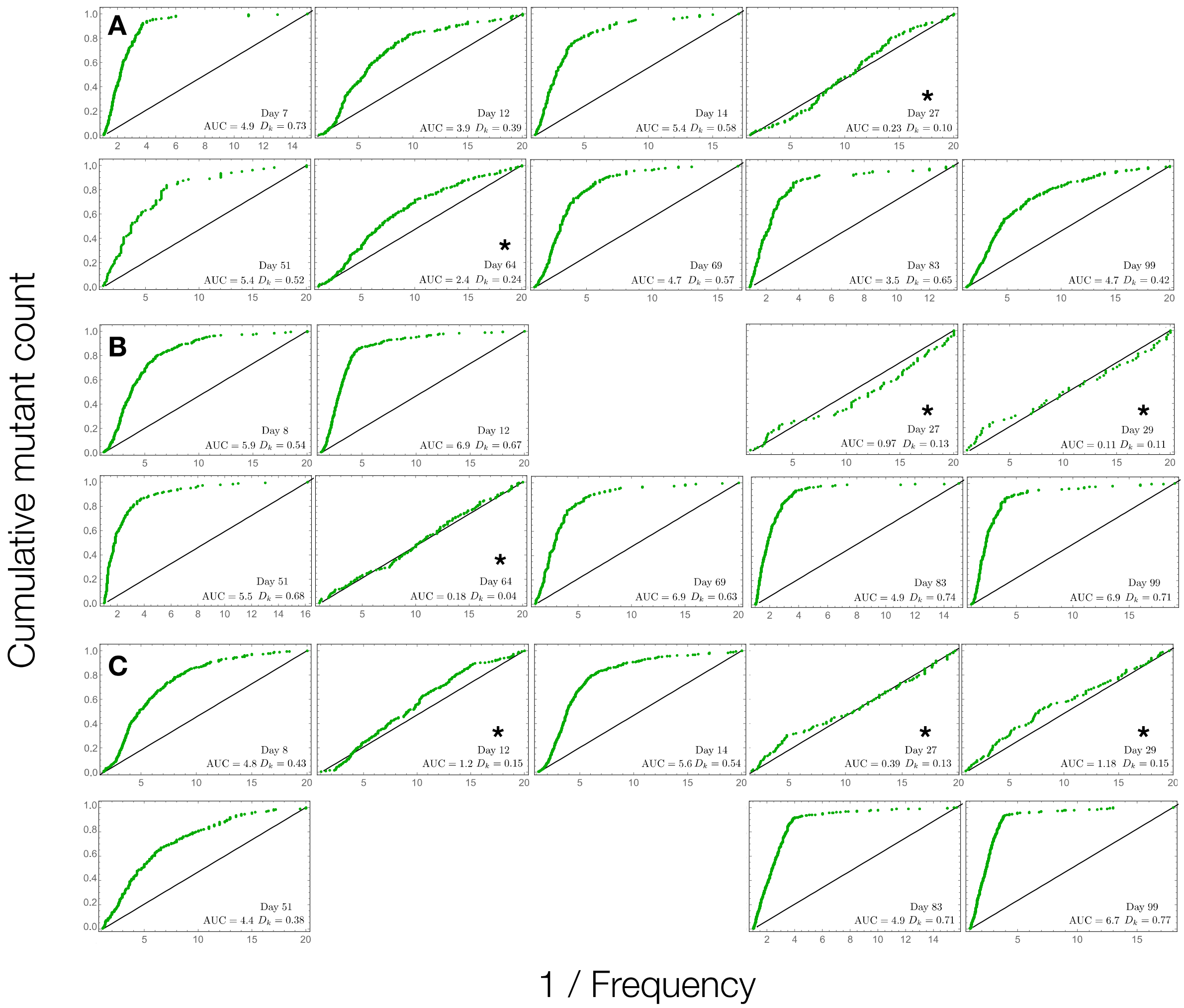

Fig. S3. Genetic diversity after selective sweeps matches expectations under neutrality. Derived allele frequencies were ordered by decreasing frequency, and normalised cumulative mutant counts are plotted as green dots against the inverse of the derived frequency. Stars denote the time points matching the Luria-Delbrück expectation under neutral population expansion (see Methods for more details). Figures (**A**), (**B**), and (**C**) correspond to replicates I, II and III, respectively. Except for the addition of day 12 in replicate III, these time points are the same as those where selective sweeps were identified based on a reduction of genetic diversity (Fig. 3B).

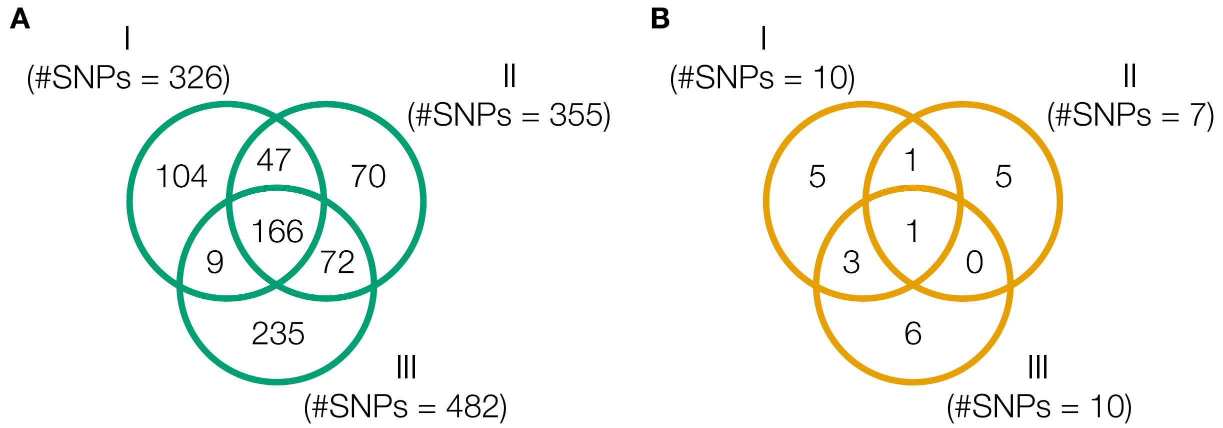

Fig. S4. Repeatable genomic change provides evidence for the action of natural selection. Venn diagrams displaying the number of mutations (SNPs) and the amount of repeatability in host (A) and virus (B) populations. A mutation was classified as repeated only when the same base change occurred at the same reference position.

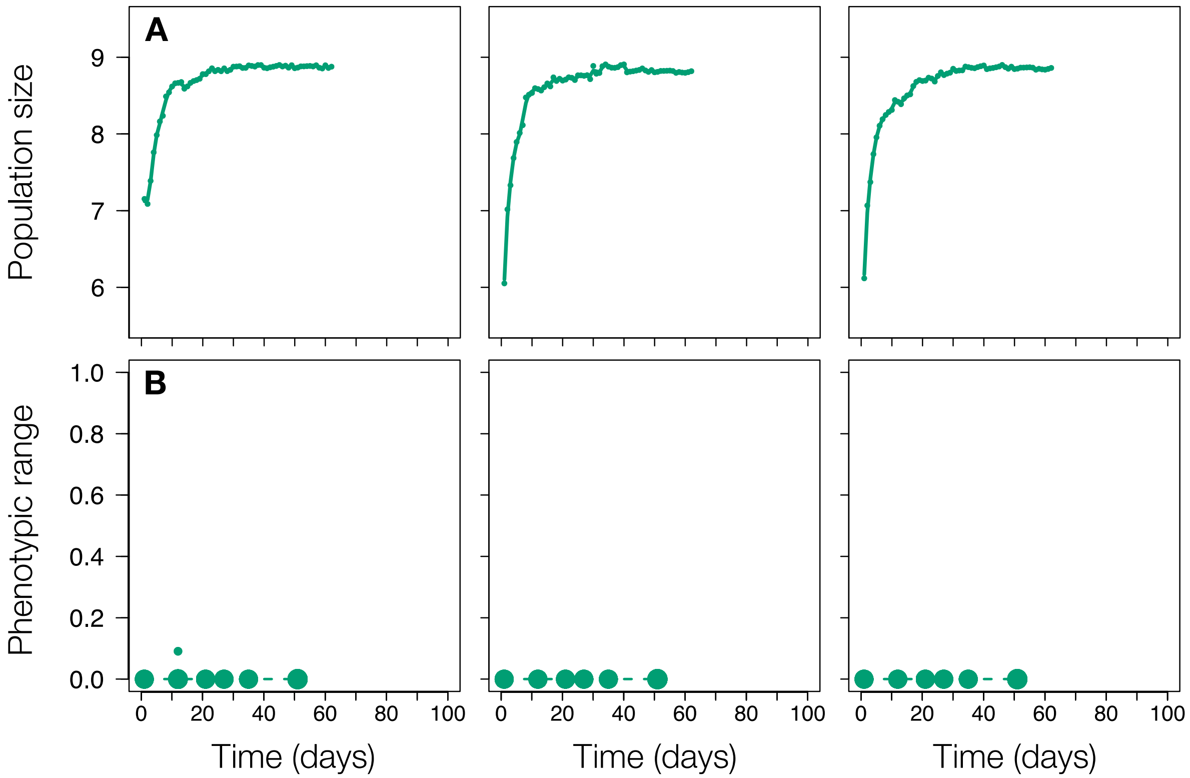

Fig. S5. Ecological change is less dynamic in the absence of species interactions. (**A**) Sizes of host populations grown in absence of the virus (dots, log10-scale), smoothed using cubic splines (lines), show exponential growth to carrying capacity. (**B**) Resistance to ancestral virus was measured for ten host clones from six time points per replicate. The size of the dots corresponds to the number of individuals with a specific phenotype. Resistance is rarely observed (one individual at day 12 of first control replicate) and not maintained.

Table S1. Sequencing and filtering statistics indicate the reliability of the genomic datasets. (A) Average depth of coverage for each time point ('A' stands for ancestral isogenic population) per reference position for host populations after quality control and read alignment. DNA was always extracted for pairs of adjacent days, and sequencing libraries were prepared for the leg (member of each pair) with the highest concentration of DNA; this is why sometimes two days are mentioned per column (e.g. 7 & 8). Because DNA was extracted directly from chemostat samples, coverage per time point is correlated to the ratio of host-to-virus particles present at the time of sampling (see Methods for details). Struck through entries were removed from analysis because of low coverage. Across replicates and time points, virus coverage ranged from 1152X to 60411X (replicate I day 83 and replicate 3 day 21, respectively), and datasets were down-sampled to 1000X for every time point (see Methods for details). (BC) Number of polymorphic loci left after each filtering step for host (B) and virus (C) datasets. Filtering steps: 1 = changes derived frequency by less than 5% (virus) or 25% (host) in the course of the experiments, 2 = within 10 bp of an indel call, 3 = above 1% derived frequency at first observation, 4 = above detection threshold at only one time point, 5 = has more than 1 (virus) or 3 (host) missing values, 6 = merged frequency trajectory of SNPs with highly correlated frequency trajectories within 1000 bp. Rows indicate the three replicates I, II, and III.

A

Day	*A*	*7<br>8*	*12*	*14<br>15*	*21*	*27*	*29*	*35<br>41*	*51*	*64*	*69<br>70*	*83*	*99*
I	39	13	33	13	~~1~~	61	~~1~~	~~0~~	11	39	15	11	40
II		33	45	~~1~~	~~1~~	125	139	~~1~~	8	83	10	13	13
III		88	168	52	~~3~~	122	154	~~4~~	28	~~0~~	~~0~~	40	56

B

Filtering step	*1*	*2*	*3*	*4*	*5*	*6*
I	10804	9232	2326	418	384	326
II	9623	8403	2177	470	454	355
IIII	13038	11126	2262	646	636	482

C

Filtering step	*1*	*2*	*3*	*4*	*5*	*6*
I	275	163	34	12	10	10
II	167	133	39	11	7	7
IIII	431	308	81	16	10	10

Table S2. Observing mutations in multiple replicates independently is unlikely under neutrality. Values reflect the number of mutations that were observed multiple times, when randomly inducing the empirical number of mutations in our experiments under stringent assumptions of neutrality, for a grid of sample sizes. Results most closely matching empirically observed overlap (fig. S4) for host (A) and virus (B) are highlighted in bold. The pool of potentially mutable sites needs to be substantially smaller than the *Chlorella variabilis* NC64A and Chlorovirus PBCV-1 reference genomes, which are respectively 42 MB and 56 kB in size and of which our approach allows us to evaluate more than 90% per replicate.

A

n_total	X_obs ± sd
1000	473 ± 7,8
1200	425 ± 8,7
1400	383 ± 9,3
1600	348 ± 9,6
1800	318 ± 9,8
**2000**	**292 ± 10,1**
2200	270 ± 10,2
2400	251 ± 10,3
2600	235 ± 10,2
2800	220 ± 10,3
3000	207 ± 10,2
5000	130 ± 9,4
10 000	67 ± 7,4

B

n_total	X_obs ± sd
20	10 ± 1,1
**50**	**5,2 ± 1,5**
100	2,8 ± 1,4
200	1,4 ± 1,1
500	0,60 ± 0,74
1000	0,30 ± 0,54

Table S3. Functional annotations of SNPs at high frequency in host populations after selective sweep at day 27. Available information on protein structure and function (GO, KOG and KEGG databases) was extracted for all repeatable mutations above 40% frequency at this time point. The "reps found" column represents in how many experimental replicates the mutation was observed, we assigned "repeatable day 27" TRUE when its frequency is repeatedly above 40% at day 27. Two out of five unique SNPs are non-synonymous. Protein 143606 has predicted histone acetyltransferase activity, which potentially affects expression profiles of a wide range of other genes. Like the mutation observed in this gene, most of the other SNPs are not only repeatable, but consistently above 40% frequency at day 27; annotation information however is limited.

replicate	chromo-some	position	reps found	repeatable day 27	predicted effect	proteinID	Predicted protein	Cellular function (kogClass)
I	6	624455	3	TRUE	non-synonymous	143606	Histone acetyltransferase activity	Transcription
I	25	504343	3	TRUE	intergenic	NA	NA	NA
II	19	680125	2	TRUE	synonymous	36653	NA	NA
II	25	504343	3	TRUE	intergenic	NA	NA	NA
II	28	201721	2	TRUE	intergenic	140025	Ubiquitin-protein ligase	NA
III	6	624455	3	TRUE	non-synonymous	143606	Histone acetyltransferase activity	Transcription
III	19	680125	2	TRUE	synonymous	36653	NA	NA
III	25	504343	3	TRUE	intergenic	NA	NA	NA
III	28	201721	2	TRUE	intergenic	140025	Ubiquitin-protein ligase	NA
III	140	1285	2	FALSE	non-synonymous	NA	NA	NA

Table S4. Functional annotations of SNPs at high frequency in host populations after selective sweep at day 64. Available information on protein structure and function (GO, KOG and KEGG databases) was extracted for all repeatable mutations above 40% frequency at this time point. The "reps found" column represents in how many experimental replicates the mutation was observed, we assigned "repeatable day 64" TRUE when its frequency is repeatably above 40% at day 64. Nine out of fifteen unique SNPs are non-synonymous. Eight are in genes encoding proteins involved in metabolism, transporting lipids, amino acids or nucleotides, and two others have a putative role in regulation of gene expression (chromatin structure and histone acetyltransferase). Though all of these potentially alter cell wall composition, no obvious candidate stands out.

replicate	chromo-some	position	reps found	repeatable day 64	predicted effect	proteinID	Predicted protein	Cellular function (kogClass)
I	2	770986	3	FALSE	non-synonymous	49863	NA	Chromatin structure and dynamics
I	3	1038582	3	FALSE	non-synonymous	140596	NA	NA
I	6	247546	3	TRUE	non-synonymous	143495	NA	NA
I	6	247548	2	FALSE	synonymous	143495	NA	NA
I	6	624455	3	TRUE	non-synonymous	143606	Histone acetyltransferase	Transcription
I	10	1373395	3	FALSE	non-synonymous	23149	Dephospho-CoA kinase	Coenzyme transport and metabolism
I	16	1053076	3	FALSE	non-synonymous	58484	Predicted lipase/calmodulin-binding heat-shock protein	Lipid transport and metabolism
I	19	683581	3	FALSE	synonymous	36653	NA	NA
I	32	280834	3	TRUE	non-synonymous	56405	Acyl-CoA synthetase	Lipid transport and metabolism
II	6	247546	3	TRUE	non-synonymous	143495	NA	NA
II	6	624455	3	TRUE	non-synonymous	143606	Histone acetyltransferase	Transcription
II	8	814819	2	FALSE	synonymous	144896	Acyl-CoA synthetase	Lipid transport and metabolism
II	10	4355	2	FALSE	synonymous	52216	Argininosuccinate synthase	Amino acid transport and metabolism
II	12	741075	2	FALSE	synonymous	24126	NA	Protein amino acid phosphorylation
II	20	806812	2	FALSE	non-synonymous	138471	NA	Lipid transport and metabolism
II	26	477054	3	FALSE	synonymous	32953	Ribonucleotide reductase	Nucleotide transport and metabolism
II	32	280834	3	TRUE	non-synonymous	56405	Acyl-CoA synthetase	Lipid transport and metabolism
II	140	1285	2	FALSE	non-synonymous	NA	NA	NA

Data file S1. Population sizes (observed and smoothed values). Population size counts for host and virus populations and smoothed values shown in Fig. 1A (.csv format)

Data file S2. Results of phenotypic assays. Growth rates for 120 host individuals in presence and absence of 11 virus populations (.csv format), used to calculate resistance and infection ranges in Fig. 1B.

Data file S3. Filtered derived allele frequencies. Derived allele frequency of SNPs meeting our filtering criteria, plotted in Fig. 4AB (.csv format).
